# The 2024 *Nucleic Acids Research* database issue and the online molecular biology database collection

**DOI:** 10.1093/nar/gkad1173

**Published:** 2023-11-30

**Authors:** Daniel J Rigden, Xosé M Fernández

**Affiliations:** Institute of Systems, Molecular and Integrative Biology, University of Liverpool, Crown Street, Liverpool L69 7ZB, UK; IQVIA Ltd., The Point, 37 North Wharf Road, London W2 1AF, UK

## Abstract

The 2024 *Nucleic Acids Research* database issue contains 180 papers from across biology and neighbouring disciplines. There are 90 papers reporting on new databases and 83 updates from resources previously published in the Issue. Updates from databases most recently published elsewhere account for a further seven. Nucleic acid databases include the new NAKB for structural information and updates from Genbank, ENA, GEO, Tarbase and JASPAR. The Issue's Breakthrough Article concerns NMPFamsDB for novel prokaryotic protein families and the AlphaFold Protein Structure Database has an important update. Metabolism is covered by updates from Reactome, Wikipathways and Metabolights. Microbes are covered by RefSeq, UNITE, SPIRE and P10K; viruses by ViralZone and PhageScope. Medically-oriented databases include the familiar COSMIC, Drugbank and TTD. Genomics-related resources include Ensembl, UCSC Genome Browser and Monarch. New arrivals cover plant imaging (OPIA and PlantPAD) and crop plants (SoyMD, TCOD and CropGS-Hub). The entire Database Issue is freely available online on the *Nucleic Acids Research* website (https://academic.oup.com/nar). Over the last year the *NAR* online Molecular Biology Database Collection has been updated, reviewing 1060 entries, adding 97 new resources and eliminating 388 discontinued URLs bringing the current total to 1959 databases. It is available at http://www.oxfordjournals.org/nar/database/c/.

## New and updated databases

The 31st *Nucleic Acids Research* database issue, in customary fashion, ranges broadly across biology with a total of 180 papers. Last year's recent record of 90 new databases is matched this year (Table 1) while 83 papers report developments from resources previously covered by *NAR*. Seven further papers provide updates on databases most recently published elsewhere (Table 2). As usual, the Issue leads off with reports from the major database providers at the European Bioinformatics Institute (EBI), the U.S. National Center for Biotechnology Information (NCBI), and the National Genomics Data Center (NGDC) in China (1–3). This year they are joined by an article from the SIB Swiss Institute of Bioinformatics (4). The following papers on individual databases are, as usual, grouped into categories: (i) nucleic acid sequence and structure, transcriptional regulation; (ii) protein sequence and structure, proteomics; (iii) metabolic and signalling pathways, enzymes and networks; (iv) genomics of viruses, bacteria, protozoa and fungi; (v) genomics of human and model organisms plus comparative genomics; (vi) human genomic variation, diseases and drugs; (vii) plants and (viii) other topics. As usual, many papers defy easy categorisation and so it is recommended that readers browse the full list.

**Table 1.** Descriptions of new databases in the 2023 NAR Database issue

Database name	URL	Short description
A3D Model Organism Database	http://biocomp.chem.uw.edu.pl/A3D2/MODB	Aggregation predictions for model organism proteomes
ABC-HuMi	https://www.ccb.uni-saarland.de/abc_humi/	Biosynthetic Gene Clusters in the Human Microbiome
ADC-DB	https://idrblab.org/adcdb/	Antibody-drug conjugates
AgeAnnoMO	https://relab.xidian.edu.cn/AgeAnnoMO/#/	Multi-omics of animal ageing
AGIDB	https://agidb.pro/	Animal Genotype Imputation DataBase
AnimalMetaOmics	https://yanglab.hzau.edu.cn/animalmetaomics#	Multi-omics of animal microbiomes
ATLAS	https://www.dsimb.inserm.fr/ATLAS	ATLAS of protein dynamics
BioExcel-CV19	https://bioexcel-cv19.bsc.es/#/	Molecular Dynamics of Covid-19 proteins
BioKA	https://ngdc.cncb.ac.cn/bioka	Biomarker Knowledgebase for Animals
CancerProteome	http://bio-bigdata.hrbmu.edu.cn/CancerProteome	The Cancer Proteome
CASpedia	http://caspedia.org	Class 2 CRISPR associated enzymes,
CDS-DB	http://cdsdb.cloudna.cn/	Cancer Drug-induced gene expression Signature DataBase
CellCommuNet	http://www.inbirg.com/cellcommunet/	Cell−cell communication analysis from scRNAseq
CellSTAR	https://idrblab.org/cellstar	Single-cell Transcriptomic Annotation Resources
cfOmics	https://cfomics.ncRNAlab.org/	Cell-free multi-omics
ClinicalOmicsDB	http://trials.linkedomics.org/	Transcriptomics and cancer clinical trials
COLOCdb	https://ngdc.cncb.ac.cn/colocdb	Cross-model colocalization for traits and phenotypes
COV2Var	http://biomedbdc.wchscu.cn/COV2Var/	SARS-CoV-2 genetic variation and consequences
CropGS-Hub	https://iagr.genomics.cn/CropGS/	Crop Genotypes and Phenotypes
CROST	https://ngdc.cncb.ac.cn/crost	Comprehensive Repository Of Spatial Transcriptomics
dbAPIS	https://bcb.unl.edu/dbAPIS	database of Anti-Prokaryotic Immune System genes
DDA	http://www.bioinformaticspa.com/DDA/	DNA Damage Atlas
DiSignAtlas	http://www.inbirg.com/disignatlas/	Disease signatures based on bulk and single-cell transcriptomics
DRMref	https://ccsm.uth.edu/DRMref/	Cancer drug resistance mechanisms from single cell data
EndoQuad	https://EndoQuad.chenzxlab.cn/	Experimental and predicted DNA G-quadruplexes
eRNAbase	http://bio.liclab.net/eRNAbase/index.php	Enhancer RNA (eRNA) in human and mouse
FL-circAS	https://cosbi.ee.ncku.edu.tw/FL-circAS/	Full-length sequences and splicing of circular RNAs
FLIBase	http://www.flibase.org	Full length isoforms in cancers and normal tissues
FusionNeoAntigen	https://compbio.uth.edu/FusionNeoAntigen	Fusion gene-specific neoantigens
FusionPDB	https://compbio.uth.edu/FusionPDB	Fusion protein annotation database
gcPathogen	https://nmdc.cn/gcpathogen/	Global Catalogue of Pathogens
GDPF	http://bioinfo.qd.sdu.edu.cn/GDPF/	Global Distribution of Protein Families
HALL	https://ngdc.cncb.ac.cn/hall/index	Human Aging and Longevity Landscape
HervD Atlas	https://ngdc.cncb.ac.cn/hervd/	Human endogenous retroviruses-disease associations
HHCDB	http://hhcdb.edbc.org/	Human heterochromatin regions
IMG/PR	https://img.jgi.doe.gov/pr	Plasmid sequences
IMP	https://www.bic.ac.cn/IMP	Integrated Medicinal Plantomics
iNClusive	https://non-canonical-aas.biologie.uni-freiburg.de/	Non-canonical amino acids
M2OR	https://m2or.chemsensim.fr/	Molecules to Olfactory Receptors
MACC	http://macc.badd-cao.net/	Metabolite-Associated Cell Communications
MAJIQlopedia	https://majiq.biociphers.org/majiqlopedia/	Human splice junctions
MAMI	https://bioinfo.biols.ac.cn/mami/	Microbiome Atlas of Mothers and Infants
MethMarkerDB	https://methmarkerdb.hzau.edu.cn/	Cancer DNA Methylation Biomarker
mHapBrowser	http://mhap.sibcb.ac.cn/	DNA Methylation Haplotype Browser
MINRbase	http://cm.jefferson.edu/MINRbase/	rRNA-derived fragments (rRFs)
MultifacetedProtDB	https://multifacetedprotdb.biocomp.unibo.it/	Proteins with multiple functions
ncRNADrug	http://www.jianglab.cn/ncRNADrug	ncRNAs targeted by drugs or linked to drug resistance
NMPFamsDB	http://www.nmpfamsdb.org/ or https://bib.fleming.gr/NMPFamsDB	Novel Metagenome Protein Families
NAKB	https://nakb.org	Nucleic Acid Knowledgebase
Open Genes	https://open-genes.org/	Human genes associated with aging and longevity
OPIA	https://ngdc.cncb.ac.cn/opia/	Open Plant Image Archive
P10K	https://ngdc.cncb.ac.cn/p10k/	Protist 10000 Genomes Project
PGS-depot	http://www.pgsdepot.net	Polygenic risk scores
PhageScope	https://phagescope.deepomics.org	Bacteriophages
PharmGWAS	https://ngdc.cncb.ac.cn/pharmgwas	GWAS for drug repurposing
PigBioBank	http://pigbiobank.farmgtex.org	Pig genotypes and phenotypes
PLAbDab	https://opig.stats.ox.ac.uk/webapps/plabdab/	Patent and Literature Antibody Database
PlantPAD	http://plantpad.samlab.cn	Plant image phenomics and disease
PMhub	https://pmhub.org.cn	Plant Metabolite hub
PPGR	https://ngdc.cncb.ac.cn/ppgr/	Resource for Perennial Plant Genomes and Regulation
PRMD	http://bioinformatics.sc.cn/PRMD	Plant RNA Modification Database
RAVAR	http://www.ravar.bio	RAre Variant Association Repository
RefMetaPlant	https://www.biosino.org/RefMetaDB/	Reference Metabolome Database for Plants
Ribocentre-switch	https://riboswitch.ribocentre.org	Riboswitches: Sequence, Structure, Function and Applications
RVdb	http://rvdb.mgc.ac.cn	Rhinovirus database
SCAN	http://scanatlas.net	Spatiotemporal Cloud Atlas for Neural cells
SCAR	http://8.142.154.29/SCAR2023	Single-cell and Spatially-resolved CAncer Resources
scGRN	https://bio.liclab.net/scGRN/	Single-cell gene regulatory networks in human and mouse
scPlantDB	https://biobigdata.nju.edu.cn/scplantdb	Plant scRNA-seq datasets
scQTLbase	http://bioinfo.szbl.ac.cn/scQTLbase	Single cell QTLs
scATAC-Ref	https://bio.liclab.net/scATAC-Ref/	Single cell ATAC-seq data
scTEA-db	http://www.scTEA-db.org	single cell-based Terminal Exon Annotation database
SilkMeta	http://silkmeta.org.cn	Silkworm pan-genome and multi-omic database
SingPro	http://idrblab.org/singpro/	Single cell proteomics
SLKB	https://slkb.osubmi.org	Synthetic lethality knowledge base
SORC	http://bio-bigdata.hrbmu.edu.cn/SORC	Spatial Omics Resource in Cancer
SoyMD	https://yanglab.hzau.edu.cn/SoyMD/#/	Soybean multi-omics
SPDB	https://scproteomicsdb.com/	Single cell proteomics
SPIRE	https://spire.embl.de	Searchable Planetary-scale mIcrobiome REsource
StemDriver	http://biomedbdc.wchscu.cn/StemDriver/	Hematopoietic stem cell differentiation by scRNAseq
STOmicsDB	https://db.cngb.org/stomics/	Spatial transcriptomics
TargetGene	https://ngdc.cncb.ac.cn/targetgene/	Target genes for human genetic variants
TCOD	https://ngdc.cncb.ac.cn/tcod	Tropical Crop omics Database
Telobase	http://cfb.ceitec.muni.cz/telobase/	Telomere sequences
TETSS	http://xozhanglab.com/TETSS	Transposon (TE)-derived transcription start sites
TheMarker	https://idrblab.org/themarker	Therapeutic biomarkers
UniTmp	http://www.unitmp.org/	Transmembrane protein structures and topologies
UTexas Aptamer Database	https://sites.utexas.edu/aptamerdatabase/	Aptamers
VarEPS-influ	https://nmdc.cn/influvar/	Influenza virus variation risk evaluation system
ZEBRA	https://www.ccb.uni-saarland.de/brain_atlas	Brain gene expression at single cell resolution

**Table 2.** Updated descriptions of databases most recently published elsewhere

Database name	URL	Short description
BATMAN-TCM	http://bionet.ncpsb.org.cn/batman-tcm/	Bioinformatics Annotation daTabase for Molecular mechANism of Traditional Chinese Medicine
circAtlas	https://ngdc.cncb.ac.cn/circatlas/	Vertebrate circular RNAs
circRNADisease	http://cgga.org.cn/circRNADisease/	Circular RNA and disease
m7GHub	http://www.rnamd.org/m7GHub2	mRNA N7-methylguanosine (m7G) sites
OrthoMAM	https://orthomam.mbb.cnrs.fr/	Curated alignments of mammalian orthologs
TPIA	http://tpia.teaplants.cn	Tea Plant Information Archive
TFcheckpoint	https://www.tfcheckpoint.org/index.php	Transcription factors from human, mouse, rat

The ‘Nucleic acid databases’ section contains a report on a major new repository of nucleic acid structural information, named NAKB (5). Updated weekly, this resource is the successor of the Nucleic Acid Database (6) and provides new annotations, visualisations, search options and database links for Protein Data Bank (PDB) (7) structures that contain nucleic acids. Other interesting new databases cover specific classes of sequence: Ribocentre-switch covers sequence, structure, function and applications of riboswitches (8), the UTex Aptamer Database (9) captures literature on aptamers, notably harnessing undergraduate power to increase curation capacity, and TeloBase covers telomere sequences across the tree of life, mining both the literature and sequence databases (10). For sequence information in general, the INSDC consortium (11) members all report updates (12–14): perhaps the most notable is the DDBJ paper which emphasises how their activities increasingly reach beyond nucleic acid sequences, most recently to metabolomics with the establishment of the MetaboBank (14). Elsewhere, two new databases cover full-length sequences from long read technologies. FLIBase (15) covers isoforms in normal and cancerous human tissues, while FL-circAS focuses specifically on circular RNAs and their splice forms (16). They are joined, for the first time in *NAR*, by the popular circAtlas for vertebrate circular RNAs reporting a tripling of content, inclusion of clinical cancer samples and, importantly, adoption of a standardised circRNA nomenclature (17). Specific epitranscriptomic modifications are covered by updates from m6A-Atlas (18) and m7GHub (19) while RMBase (20) covers data related to 73 distinct modifications in 62 species, and the general database of RNA modifications, pathways and compounds MODOMICS adds novel modifications, both those seen experimentally in ribosome structures and artificial modifications used as experimental tools (21).

In gene expression and regulation, the foundational NCBI Gene Expression Omnibus (GEO) database returns (22) after a gap of 11 years to reflect on the gradual replacement of array-based data with next-generation sequence information, and the growing proportion of studies with a single-cell focus (also very visible in this Issue, see below). The venerable Tarbase, for miRNA-gene interactions, reports a six-fold increase in content since the previous version and new data types such as virally encoded miRNAs and interactions leading to target-directed miRNA degradation (23). Finally, major databases of transcription factor (TF) binding sites publish updates: HOCOMOCO reports its latest collection of carefully processed and curated human and mouse TF motifs (24) while JASPAR celebrates its 20th anniversary with useful increases in curated content across taxa and a reflection on the challenges to be addressed as the spread of deep learning methods across biology continues (25).

The protein section contains a ‘Breakthrough Article’ in the form of the NMPFamsDB paper (26). This database contains the results of mining metagenome sequences for novel families that are unrelated to those in the well-known Pfam database (27) or reference proteomes. Each entry in this veritable gold mine is comprehensively presented as a sequence alignment and (in most cases) an AlphaFold 2 model (28), alongside genome context, taxonomic mapping and geographical distribution. Equally momentous is an update article from the AlphaFold Protein Structure Database (29) which now covers around 90% of the sequence space in the UniProt database (30) with state of the art protein models. A range of improvements to the website cover, for example, a new sequence search by BLAST (31), easy access to different clusters of models, and improved interaction with content, both coordinates and the crucial PAE. Another update from the EBI covers EMDB, the Electron Microscopy Data Bank which reports continued experimental growth, responses to rapid technological change, and plans for validating data and metadata (32).

Elsewhere among protein-related databases, interesting new arrivals include inCLusive (33) capturing information on over 400 non-canonical amino acids and their incorporation into proteins, and MultifacetedProtDB (34) with >1000 multi-functional aka moonlighting proteins. Two new databases SingPro (35) and SPDB (36) capture single-cell proteomics data and each offer sophisticated visualisation and analytical options. Elsewhere a pair of new databases cover Molecular Dynamics simulations, surely a significant growth area in the post-AlphaFold 2 era. BioExcel-CV19 (37) majors on simulations of Covid19 proteins but establishes an infrastructure designed to be reused elsewhere, while ATLAS (38) covers simulations of almost 1400 proteins selected to span most of structure space as defined by high level X-class domains in the ECOD hierarchical classification (39). Another notable new name is UniTmp which brings together a number of well-known resources for transmembrane proteins, including previous Database Issue regulars (40, 41), into an integrated one-stop shop (42). A trio of well-known returning databases cover proteins that are not conventionally folded. The update from PED (43), covering ensembles of intrinsically disordered proteins (IDPs), reports a tripling of size and the new inclusion of purely computationally generated ensembles from eg. Machine Learning methods. It is complemented by a report from DisProt (44) which emphasises the importance of adoption of community standards in the protein disorder field (45) and reports an interesting bi-directional relationship with Gene Ontology (46) whose terms are used for annotation, but whose vocabulary is also expanded with input from the DisProt team. IDPs commonly contain linear interaction motifs of the kind documented by ELM (47) which reports interesting new motifs in eg RNA processing and protein degradation, and the news that ELM is now integrated into InterPro (48). Finally, two databases returning after 10 or more years cover protein-ligand interactions observed in the PDB: BioLiP (49) returns with its analysis of biologically relevant bound ligands, while MESPEUS (50) focuses on bound metals, usefully including sites that lie at crystal lattice interfaces, and offering pan-PDB coordination number distributions and measures of deviation from ideal geometry.

In the section for metabolism, signalling and enzymes, two new databases focus on cell–cell communications. CellCommuNet (51) exploits single cell RNAseq information to illuminate human and mouse cell-cell communication networks in health and disease while MACC (52) focuses on the role of metabolites and offers a bespoke visualisation tool for metabolite-cell regulatory networks. In the field of gene clusters, the popular antiSMASH database contributes an update (53) and is joined by the new ABC-HuMi (54) which considers distributions across different human microbiomes associated with different tissues. Metabolic pathways in general are amply covered by updates from three important returning databases: Reactome (55) now covers more than 11 000 human proteins (around 56%) and the paper discusses approaches to cover the remainder; WikiPathways (56) reaches almost 2000 pathways and 27 species, and benefits from an intriguing new tool to recognise terms in static pathway figures from the literature (pfocr.wikipathways.org); and PathBank (57) reports massively increased content, better pathway images and pathway enrichment tools. Finally, the major metabolomics resource Metabolights contributes an update (58) reporting its growth six-fold in studies since the last report, but also showcasing the MetaboLights Labs for exposing and distributing relevant computational workflows.

Viruses are particularly well represented in the next section they share with microbes. New arrivals VarEPS-influ (59), RVdb (60) and COV2Var (61) focus respectively on influenza, with an especial aim to understand and evaluate the risks of future epidemics; on rhinovirus, responsible for many common colds and linked to more serious conditions; and on SARS-CoV-2 genetic variation among 13 billion genome sequences. Returning virus databases include PhageScope (62) which covers almost a million comprehensively annotated bacteriophage sequences, and ViralZone (63) the beautifully illustrated educational virology resource. Eukaryotes are covered firstly by the interesting new P10K database (64), the home of the protist 10 000 Genomes Project. This has already covered almost half of protist orders by using single cell techniques to avoid difficulties in culturing, and has revealed intriguing variability in genetic codes among ciliates. There are also the returning VEuPathDB (65) for eukaryotic pathogens and hosts which adds new data types—AlphaFold 2 structure predictions and single cell transcriptomics; and UNITE (66), the extremely popular resource for eukaryote molecular identification which expands from a fungal focus to cover all eukaryotes. Finally, two new databases map sequence data geographically: SPIRE (67) includes curated global metagenomes annotated with habitat and geography, while GDPF (68) maps prokaryotic protein families in terms of global distribution and environmental abundance.

In the human, model organism and comparative genomics section, no fewer than four papers cover aging. Open Genes (69), a new arrival, majors on easy access to carefully curated and scored (meta)data of diverse types—gene expression, methylation, protein activity, genetic variants and so on—in both human and model organism orthologs. The popular returning resource HAGR (70), brings updates to its set of six database components, and two new databases, AgeAnnoMO (71) and HALL (72) each report impressive coverage of multi-omics data relating to ageing and longevity. Elsewhere the trends towards single cell and spatial expression data are again evident. For example, CellSTAR (73) is a new database for single-cell transcriptomics which offers annotation of hundreds of different cell types and markers across 18 species and 139 tissues. Spatial transcriptomics is covered by two new databases, STOmicsDB (74) and CROST (75) which each cover a range of species, tissues and diseases, and offer a superb range of visualisation options. Notably, the latter offers a cancer module integrating other kinds of relevant information such as copy number variation and epigenomics. In comparative genomics, two cornerstone resources provide updates. Ensembl (76) reports efforts on its Rapid Release site to provide quick annotations for outputs of biodiversity projects such as the Darwin Tree of Life; work in the area of food security, from livestock to ‘orphan crops’ to pests; and continued engagement with users around new features on its new Beta website. An update on the UCSC Genome Browser (77) reports on new species browsers, new data tracks for model species, and even new forms of data visualisation such as sequence logos. Finally Monarch updates (78) on its efforts to map genes, phenotypes and diseases across model organisms. Their Knowledge Graph is suitable for Machine Learning methods, both to extract new biological insights but also for querying by their new ChatGPT plugin. Monarch is far from the only database looking at such large language models to summarise and present content to the user: it will clearly be important to understand both the opportunities and the risks, and to share best practice in the area.

As usual, cancer has a strong presence in the section on human genomic variation, diseases and drugs. COSMIC, the foundational database of variants and clinical data, returns to report new features such as a mutational signatures catalogue and an Actionability section to record clinical trials linked to particular variants (79). A valuable pair of new databases, CDS-DB (80) and ClinicalOmicsDB (81), focus on transcriptomics responses to cancer drug treatment. Another pair of new databases focus on fusion proteins, the result of gene fusion events: FusionPDB (82) catalogues and annotates fusion protein sequences, additionally taking some through to AlphaFold 2 modelling and small molecule screening; while FusionNeoAntigen (83) predicts neoantigens that span fusion gene breakpoints, testing their putative interaction with HLA molecules by explicit docking studies. A further duo of new arrivals, SCAR (84) and SORC (85) each cover spatial omics in cancer and offer similarly comprehensive resources for single cell transcriptomics data, biomarkers, cell-cell communications and so on. For drug targets, two major resources submit updates: the Therapeutic Target Database (86) features a major new focus on methods for predicting druggability while DGIdb (87) has new data sources, a new interface and improved FAIRness (i.e. findability, accessibility, interoperability and reusability). Major drug and chemical databases contributing updates are DrugBank (88), which reports a range of improvements including inclusion of mass spectrometry data and concise graphics illustrating drug mechanisms and metabolic pathways; and ChEMBL (89) which now covers 2 million unique compounds and 20 million bioactivity measurements. A number of databases address drug behaviour in the body including returning databases VARIDT (90) and INTEDE (91) for drug transporters and metabolism respectively. The former now covers the influence of microbiota, post-translational modifications and differential expression on drug transporters while the latter offers superbly detailed and -presented information on drug metabolic pathways and their intermediates. The new DRMref (92) uses scRNA-seq to identify drug resistance-related molecular mechanisms across different drugs, cell types and cancers.

Variant interpretation remains a key scientific challenge and two returning databases publish updates. The very popular CADD (93) reports improved performance from addition of new component scores, including from natural language methods, while VarCards (94) is designed especially to support clinical interpretation of variants by genetic counsellors. A trio of returning databases linking ncRNA to disease publish updates: HMDD (95) grows substantially and covers new categories of data such as exosomal or virus-encoded miRNAs; LncRNADisease (96) doubles in size and now distinguishes between causative and correlative disease relationships; and CircRNADisease (97) grows 20-fold in content and includes new information, for example on circRNA mutations and cancer. Finally, two major ontologies report updates in this section: the Human Disease Ontology celebrates 20 years (98) with a new Knowledgebase and provides insights into the exhaustive community discussions driving expansion of the resource; while the Human Phenotype Ontology reports similar collaborative efforts, as well as impressive progress in translating the ontology into other languages (99).

The plant database section features a particular rich crop of new databases. Two cover plant imaging: OPIA (100) has half a million images and links genomic rice and wheat data to phenotypic i-traits (characteristics from image analysis such as plant height); while the PlantPAD paper (101) offers interesting cases studies relating to their image data, such as machine learning for automated disease diagnosis. Other new resources cover crop plants: SoyMD (102) offers comprehensive multi-omics data on soybean to aid breeding efforts; TCOD (103) offers a variety of data related to 15 tropical crops; and CropGS-Hub (104) links genotype and phenotype in seven major crops. Elsewhere, PPGR (105) covers multi-omics and related analytical tools for the study of woody perennial plants, including 60 genome assemblies. A pair of new databases cover plant metabolomics: RefMetaPlant (106) constructs a reference metabolome from mass spectrometry measurements of more than 150 plants across the five major phyla while PMhub (107) notably includes genomic and transcriptomic information to enable genetic analysis of metabolites.

The final section features databases not readily accommodated elsewhere. LIPID MAPS returns after a long absence (108) to support lipidomics research with manual curation of literature, a defined lipid classification, and a renewed focus on FAIR principles. The UniLectin update (109) reports a new focused HumanLectome module while the popular Vesiclepedia database (110) now extends its coverage to new classes of extracellular particle such as exomeres and supermeres.

## NAR online molecular biology database collection

Since the previous update, our *NAR* online Molecular Biology Database Collection (freely available at http://www.oxfordjournals.org/nar/database/c/) has been extensively updated, reviewing 1060 entries. 97 new resources listed in this issue were added to the database and 388 obsolete entries have been removed accordingly. Further curation efforts were made reviewing recent updates and user feedback bringing the total to 1959 databases in this new update. We encourage authors to submit their updates to XMF at xose.m.fernandez@gmail.com in plain text, ideally according to the template found in http://www.oxfordjournals.org/nar/database/summary/1.

In times of fast-moving technological change, we are keen to hear from the community about any changes we could make to the database collection to enhance its value. Feedback can be sent to nardatabase@gmail.com.

## References

[1] Thakur M. , Buniello A., Brooksbank C., Gurwitz K.T., Hall M., Hartley M., Hulcoop D.G., Leach A.R., Marques D., Martin M. et al. EMBL’s European Bioinformatics Institute (EMBL-EBI) in 2023. Nucleic Acids Res. 2023; 10.1093/nar/gkad1088. PMC10767983 38015445

[2] Sayers E.W. , Beck J., Bolton E.E., Brister J.R., Chan J., Comeau D.C., Connor R., DiCuccio M., Farrell C.M., Feldgarden M. et al. Database resources of the National Center for Biotechnology Information. Nucleic Acids Res. 2023; 10.1093/nar/gkad1044. PMC10767890 37994677

[3] CNCB-NGDC Members and Partners Database Resources of the National Genomics Data Center, China National Center for Bioinformation in 2024. Nucleic Acids Res. 2023; 10.1093/nar/gkad1078. PMC10767964 38018256

[4] SIB Swiss Institute of Bioinformatics RDF Group Members The SIB Swiss Institute of Bioinformatics Semantic Web of data. Nucleic Acids Res. 2023; 10.1093/nar/gkad902. PMC10767860 37878411

[5] Lawson C.L. , Berman H.M., Chen L., Vallat B., Zirbel C.L. The Nucleic Acid Knowledgebase: a new portal for 3D structural information about nucleic acids. Nucleic Acids Res. 2023; 10.1093/nar/gkad957. PMC10767938 37953312

[6] Coimbatore Narayanan B. , Westbrook J., Ghosh S., Petrov A.I., Sweeney B., Zirbel C.L., Leontis N.B., Berman H.M The Nucleic Acid Database: new features and capabilities. Nucleic Acids Res. 2014; 42:D114–D122. 24185695 10.1093/nar/gkt980 PMC3964972

[7] wwPDB consortium Protein Data Bank: the single global archive for 3D macromolecular structure data. Nucleic Acids Res. 2019; 47:D520–D528. 30357364 10.1093/nar/gky949 PMC6324056

[8] Bu F. , Lin X., Liao W., Lu Z., He Y., Luo Y., Peng X., Li M., Huang Y., Chen X. et al. Ribocentre-switch: a database of riboswitches. Nucleic Acids Res. 2023; 10.1093/nar/gkad891. PMC10767811 37855663

[9] Askari A. , Kota S., Ferrell H., Swamy S., Goodman K.S., Okoro C.C., Spruell Crenshaw I.C., Hernandez D.K., Oliphant T.E., Badrayani A.A. et al. UTexas Aptamer Database: the collection and long-term preservation of aptamer sequence information. Nucleic Acids Res. 2023; 10.1093/nar/gkad959. PMC10767891 37904593

[10] Lyčka M. , Bubeník M., Závodník M., Peska V., Fajkus P., Demko M., Fajkus J., Fojtová M. TeloBase: a community-curated database of telomere sequences across the tree of life. Nucleic Acids Res. 2023; 10.1093/nar/gkad672. PMC10767889 37602392

[11] Arita M. , Karsch-Mizrachi I., Cochrane G. The international nucleotide sequence database collaboration. Nucleic Acids Res. 2021; 49:D121–D124. 33166387 10.1093/nar/gkaa967 PMC7778961

[12] Sayers E.W. , Cavanaugh M., Clark K., Pruitt K.D., Sherry S.T., Yankie L., Karsch-Mizrachi I. GenBank 2024 update. Nucleic Acids Res. 2023; 10.1093/nar/gkad903. PMC10767886 37889039

[13] Yuan D. , Ahamed A., Burgin J., Cummins C., Devraj R., Gueye K., Gupta D., Gupta V., Haseeb M., Ihsan M. et al. The European Nucleotide Archive in 2023. Nucleic Acids Res. 2023; 10.1093/nar/gkad1067. PMC10767888 37956313

[14] Ara T. , Kodama Y., Tokimatsu T., Fukuda A., Kosuge T., Mashima J., Tanizawa Y., Tanjo T., Ogasawara O., Fujisawa T. et al. DDBJ update in 2023: the MetaboBank for metabolomics data and associated metadata. Nucleic Acids Res. 2023; 10.1093/nar/gkad1046. PMC10767850 37971299

[15] Shi Q. , Li X., Liu Y., Chen Z., He X. FLIBase: a comprehensive repository of full-length isoforms across human cancers and tissues. Nucleic Acids Res. 2023; 10.1093/nar/gkad745. PMC10767943 37697439

[16] Chiang T.W. , Jhong S.E., Chen Y.C., Chen C.Y., Wu W.S., Chuang T.J. FL-circAS: an integrative resource and analysis for full-length sequences and alternative splicing of circular RNAs with nanopore sequencing. Nucleic Acids Res. 2023; 10.1093/nar/gkad829. PMC10767854 37823705

[17] Wu W. , Zhao F., Zhang J. circAtlas 3.0: a gateway to 3 million curated vertebrate circular RNAs based on a standardized nomenclature scheme. Nucleic Acids Res. 2023; 10.1093/nar/gkad770. PMC10767913 37739414

[18] Liang Z. , Ye H., Ma J., Wei Z., Wang Y., Zhang Y., Huang D., Song B., Meng J., Rigden D.J. et al. m6A-Atlas v2.0: updated resources for unraveling the N6-methyladenosine (m6A) epitranscriptome among multiple species. Nucleic Acids Res. 2023; 10.1093/nar/gkad691. PMC10768109 37587690

[19] Wang X. , Zhang Y., Chen K., Liang Z., Ma J., Xia R., de Magalhães J.P., Rigden D.J., Meng J., Song B. m7GHub V2.0: an updated database for decoding the N7-methylguanosine (m7G) epitranscriptome. Nucleic Acids Res. 2023; 10.1093/nar/gkad789. PMC10767970 37811871

[20] Xuan J. , Chen L., Chen Z., Pang J., Huang J., Lin J., Zheng L., Li B., Qu L., Yang J. RMBase v3.0: decode the landscape, mechanisms and functions of RNA modifications. Nucleic Acids Res. 2023; 10.1093/nar/gkad1070. PMC10767931 37956310

[21] Cappannini A. , Ray A., Purta E., Mukherjee S., Boccaletto P., Moafinejad S.N., Lechner A., Barchet C., Klaholz B.P., Stefaniak F. et al. MODOMICS: a database of RNA modifications and related information. 2023 update. Nucleic Acids Res. 2023; 10.1093/nar/gkad1083. PMC10767930 38015436

[22] Clough E. , Barrett T., Wilhite S.E., Ledoux P., Evangelista C., Kim I.F., Tomashevsky M., Marshall K.A., Phillippy K.H., Sherman P.M. et al. NCBI GEO: archive for gene expression and epigenomics data sets: 23-year update. Nucleic Acids Res. 2023; 10.1093/nar/gkad965. PMC10767856 37933855

[23] Skoufos G. , Kakoulidis P., Tastsoglou S., Zacharopoulou E., Kotsira V., Miliotis M., Mavromati G., Grigoriadis D., Zioga M., Velli A. et al. TarBase-v9.0 extends experimentally supported miRNA-gene interactions to cell-types and virally encoded miRNAs. Nucleic Acids Res. 2023; 10.1093/nar/gkad1071. PMC10767993 37986224

[24] Vorontsov I.E. , Eliseeva I.A., Zinkevich A., Nikonov M., Abramov S., Boytsov A., Kamenets V., Kasianova A., Kolmykov S., Yevshin I.S. et al. HOCOMOCO in 2024: a rebuild of the curated collection of binding models for human and mouse transcription factors. Nucleic Acids Res. 2023; 10.1093/nar/gkad1077. PMC10767914 37971293

[25] Rauluseviciute I. , Riudavets-Puig R., Blanc-Mathieu R., Castro-Mondragon J.A., Ferenc K., Kumar V., Lemma R.B., Lucas J., Chèneby J., Baranasic D. et al. JASPAR 2024: 20th anniversary of the open-access database of transcription factor binding profiles. Nucleic Acids Res. 2023; 10.1093/nar/gkad1059. PMC10767809 37962376

[26] Baltoumas F.A. , Karatzas E., Liu S., Ovchinnikov S., Sofianatos Y., Chen I.M., Kyrpides N.C., Pavlopoulos G.A. NMPFamsDB: a database of novel protein families from microbial metagenomes and metatranscriptomes. Nucleic Acids Res. 2023; 10.1093/nar/gkad800. PMC10767849 37811892

[27] Mistry J. , Chuguransky S., Williams L., Qureshi M., Salazar G.A., Sonnhammer E.L., Tosatto S.C., Paladin L., Raj S., Richardson L.J. et al. Pfam: the protein families database in 2021. Nucleic Acids Res. 2021; 49:D412–D419. 33125078 10.1093/nar/gkaa913 PMC7779014

[28] Jumper J. , Evans R., Pritzel A., Green T., Figurnov M., Ronneberger O., Tunyasuvunakool K., Bates R., Žídek A., Potapenko A. et al. Highly accurate protein structure prediction with AlphaFold. Nature. 2021; 596:583–589. 34265844 10.1038/s41586-021-03819-2 PMC8371605

[29] Varadi M. , Bertoni D., Magana P., Paramval U., Pidruchna I., Radhakrishnan M., Tsenkov M., Nair S., Mirdita M., Yeo J. et al. AlphaFold Protein Structure Database in 2024: providing structure coverage for over 214 million protein sequences. Nucleic Acids Res. 2023; 10.1093/nar/gkad1011. PMC10767828 37933859

[30] UniProt Consortium UniProt: the Universal Protein Knowledgebase in 2023. Nucleic Acids Res. 2023; 51:D523–D531. 36408920 10.1093/nar/gkac1052 PMC9825514

[31] Altschul S.F. , Madden T.L., Schäffer A.A., Zhang J., Zhang Z., Miller W., Lipman D.J. Gapped BLAST and PSI-BLAST: a new generation of protein database search programs. Nucleic Acids Res. 1997; 25:3389–3402. 9254694 10.1093/nar/25.17.3389 PMC146917

[32] The wwPDB Consortium EMDB—the Electron Microscopy Data Bank. Nucleic Acids Res. 2023; 10.1093/nar/gkad1019. PMC10767987 37994703

[33] Icking L.S. , Riedlberger A.M., Krause F., Widder J., Frederiksen A.S., Stockert F., Spädt M., Edel N., Armbruster D., Forlani G. et al. iNClusive: a database collecting useful information on non-canonical amino acids and their incorporation into proteins for easier genetic code expansion implementation. Nucleic Acids Res. 2023; 10.1093/nar/gkad1090. PMC10767842 37986218

[34] Bertolini E. , Babbi G., Savojardo C., Martelli P.L., Casadio R. MultifacetedProtDB: a database of human proteins with multiple functions. Nucleic Acids Res. 2023; 10.1093/nar/gkad783. PMC10767882 37791887

[35] Lian X. , Zhang Y., Zhou Y., Sun X., Huang S., Dai H., Han L., Zhu F. SingPro: a knowledge base providing single-cell proteomic data. Nucleic Acids Res. 2023; 10.1093/nar/gkad830. PMC10767818 37819028

[36] Wang F. , Liu C., Li J., Yang F., Song J., Zang T., Yao J., Wang G. SPDB: a comprehensive resource and knowledgebase for proteomic data at the single-cell resolution. Nucleic Acids Res. 2023; 10.1093/nar/gkad1018. PMC10767837 37953313

[37] Beltrán D. , Hospital A., Gelpí J.L., Orozco M. A new paradigm for molecular dynamics databases: the COVID-19 database, the legacy of a titanic community effort. Nucleic Acids Res. 2023; 10.1093/nar/gkad991. PMC10767965 37953362

[38] Vander Meersche Y. , Cretin G., Gheeraert A., Gelly J.C., Galochkina T. ATLAS: protein flexibility description from atomistic molecular dynamics simulations. Nucleic Acids Res. 2023; 10.1093/nar/gkad1084. PMC10767941 37986215

[39] Cheng H. , Schaeffer R.D., Liao Y., Kinch L.N., Pei J., Shi S., Kim B.H., Grishin N.V. ECOD: an evolutionary classification of protein domains. PLoS Comput. Biol. 2014; 10:e1003926. 25474468 10.1371/journal.pcbi.1003926 PMC4256011

[40] Kozma D. , Simon I., Tusnady G.E. PDBTM: protein Data Bank of transmembrane proteins after 8 years. Nucleic Acids Res. 2012; 41:D524–D529. 23203988 10.1093/nar/gks1169 PMC3531219

[41] Dobson L. , Lango T., Reményi I., Tusnády G.E. Expediting topology data gathering for the TOPDB database. Nucleic Acids Res. 2015; 43:D283–D289. 25392424 10.1093/nar/gku1119 PMC4383934

[42] Dobson L. , Gerdán C., Tusnády S., Szekeres L., Kuffa K., Langó T., Zeke A., Tusnády G.E. UniTmp: unified resources for transmembrane proteins. Nucleic Acids Res. 2023; 10.1093/nar/gkad897. PMC10767979 37870462

[43] Ghafouri H. , Lazar T., Del Conte A., Ku T., L. G., Consortium P.E.D., Tompa P., Tosatto S.C.E., Monzon A.M PED in 2024: improving the community deposition of structural ensembles for intrinsically disordered proteins. Nucleic Acids Res. 2023; 10.1093/nar/gkad947. PMC10767937 37904608

[44] Aspromonte M.C. , Nugnes M.V., Quaglia F., Bouharoua A., Consortium D.P., Tosatto S.C.E., Piovesan D DisProt in 2024: improving function annotation of intrinsically disordered proteins. Nucleic Acids Res. 2023; 10.1093/nar/gkad928. PMC10767923 37904585

[45] Mészáros B. , Hatos A., Palopoli N., Quaglia F., Salladini E., Van Roey K., Arthanari H., Dosztányi Z., Felli I.C., Fischer P.D. et al. Minimum information guidelines for experiments structurally characterizing intrinsically disordered protein regions. Nat. Methods. 2023; 20:1291–1303. 37400558 10.1038/s41592-023-01915-x

[46] Gene Ontology Consortium The Gene Ontology resource: enriching a GOld mine. Nucleic Acids Res. 2021; 49:D325–D334. 33290552 10.1093/nar/gkaa1113 PMC7779012

[47] Kumar M. , Michael S., Alvarado-Valverde J., Zeke A., Lazar T., Glavina J., Nagy-Kanta E., Donagh J.M., Kalman Z.E., Pascarelli S. et al. ELM-the Eukaryotic Linear Motif resource-2024 update. Nucleic Acids Res. 2023; 10.1093/nar/gkad1058. PMC10767929 37962385

[48] Paysan-Lafosse T. , Blum M., Chuguransky S., Grego T., Pinto B.L., Salazar G.A., Bileschi M.L., Bork P., Bridge A., Colwell L. et al. InterPro in 2022. Nucleic Acids Res. 2023; 51:D418–D427. 36350672 10.1093/nar/gkac993 PMC9825450

[49] Zhang C. , Zhang X., Freddolino P.L., Zhang Y. BioLiP2: an updated structure database for biologically relevant ligand-protein interactions. Nucleic Acids Res. 2023; 10.1093/nar/gkad630. PMC10767969 37522378

[50] Lin G.Y. , Su Y.C., Huang Y.L., Hsin K.Y. MESPEUS: a database of metal coordination groups in proteins. Nucleic Acids Res. 2023; 10.1093/nar/gkad1009. PMC10767821 37941148

[51] Ma Q. , Li Q., Zheng X., Pan J. CellCommuNet: an atlas of cell-cell communication networks from single-cell RNA sequencing of human and mouse tissues in normal and disease states. Nucleic Acids Res. 2023; 10.1093/nar/gkad906. PMC10767892 37850657

[52] Gao J. , Mo S., Wang J., Zhang M., Shi Y., Zhu C., Shang Y., Tang X., Zhang S., Wu X. et al. MACC: a visual interactive knowledgebase of metabolite-associated cell communications. Nucleic Acids Res. 2023; 10.1093/nar/gkad914. PMC10767829 37897362

[53] Blin K. , Shaw S., Medema M.H., Weber T. The antiSMASH database version 4: additional genomes and BGCs, new sequence-based searches and more. Nucleic Acids Res. 2023; 10.1093/nar/gkad984. PMC10767862 37904617

[54] Hirsch P. , Tagirdzhanov A., Kushnareva A., Olkhovskii I., Graf S., Schmartz G.P., Hegemann J.D., Bozhüyük K.A.J., Müller R., Keller A. et al. ABC-HuMi: the Atlas of Biosynthetic Gene Clusters in the Human Microbiome. Nucleic Acids Res. 2023; 10.1093/nar/gkad1086. PMC10767846 37994699

[55] Milacic M. , Beavers D., Conley P., Gong C., Gillespie M., Griss J., Haw R., Jassal B., Matthews L., May B. et al. The Reactome Pathway Knowledgebase 2024. Nucleic Acids Res. 2023; 10.1093/nar/gkad1025. PMC10767911 37941124

[56] Agrawal A. , Balcı H., Hanspers K., Coort S.L., Martens M., Slenter D.N., Ehrhart F., Digles D., Waagmeester A., Wassink I. et al. WikiPathways 2024: next generation pathway database. Nucleic Acids Res. 2023; 10.1093/nar/gkad960. PMC10767877 37941138

[57] Wishart D.S. , Kruger R., Sivakumaran A., Harford K., Sanford S., Doshi R., Kehrtarpal N., Fatokun O., Doucet D., Zubkowski A. et al. PathBank 2.0-the pathway database for model organism metabolomics. Nucleic Acids Res. 2023; 10.1093/nar/gkad1041. PMC10767802 37962386

[58] Yurekten O. , Payne T., Tejera N., Amaladoss F.X., Martin C., Williams M., O’Donovan C MetaboLights: open data repository for metabolomics. Nucleic Acids Res. 2023; 10.1093/nar/gkad1045. PMC10767962 37971328

[59] Shu C. , Sun Q., Fan G., Peng K., Yu Z., Luo Y., Gao S., Ma J., Deng T., Hu S. et al. VarEPS-Influ:an risk evaluation system of occurred and virtual variations of influenza virus genomes. Nucleic Acids Res. 2023; 10.1093/nar/gkad912. PMC10767863 37889020

[60] Zhao P. , Zhou S., Xu P., Su H., Han Y., Dong J., Sui H., Li X., Hu Y., Wu Z. et al. RVdb: a comprehensive resource and analysis platform for rhinovirus research. Nucleic Acids Res. 2023; 10.1093/nar/gkad937. PMC10768139 37930838

[61] Feng Y. , Yi J., Yang L., Wang Y., Wen J., Zhao W., Kim P., Zhou X. COV2Var, a function annotation database of SARS-CoV-2 genetic variation. Nucleic Acids Res. 2023; 10.1093/nar/gkad958. PMC10767816 37897356

[62] Wang R.H. , Yang S., Liu Z., Zhang Y., Wang X., Xu Z., Wang J., Li S.C. PhageScope: a well-annotated bacteriophage database with automatic analyses and visualizations. Nucleic Acids Res. 2023; 10.1093/nar/gkad979. PMC10767790 37904614

[63] De Castro E. , Hulo C., Masson P., Auchincloss A., Bridge A., Le Mercier P ViralZone 2024 provides higher-resolution images and advanced virus-specific resources. Nucleic Acids Res. 2023; 10.1093/nar/gkad946. PMC10767872 37897348

[64] Gao X. , Chen K., Xiong J., Zou D., Yang F., Ma Y., Jiang C., Gao X., Wang G., Gu S. et al. The P10K database: a data portal for the protist 10 000 genomes project. Nucleic Acids Res. 2023; 10.1093/nar/gkad992. PMC10767852 37930867

[65] Alvarez-Jarreta J. , Amos B., Aurrecoechea C., Bah S., Barba M., Barreto A., Basenko E.Y., Belnap R., Blevins A., Böhme U. et al. VEuPathDB: the eukaryotic pathogen, vector and host bioinformatics resource center in 2023. Nucleic Acids Res. 2023; 10.1093/nar/gkad1003. PMC10767879 37953350

[66] Abarenkov K. , Nilsson R.H., Larsson K.H., Taylor A.F.S., May T.W., Frøslev T.G., Pawlowska J., Lindahl B., Põldmaa K., Truong C. et al. The UNITE database for molecular identification and taxonomic communication of fungi and other eukaryotes: sequences, taxa and classifications reconsidered. Nucleic Acids Res. 2023; 10.1093/nar/gkad1039. PMC10767974 37953409

[67] Schmidt T.S.B. , Fullam A., Ferretti P., Orakov A., Maistrenko O.M., Ruscheweyh H.J., Letunic I., Duan Y., Van Rossum T., Sunagawa S. et al. SPIRE: a Searchable, Planetary-scale mIcrobiome REsoure. Nucleic Acids Res. 2023; 10.1093/nar/gkad943. PMC10767986 37897342

[68] Pan Z. , Li D.D., Li P., Geng Y., Jiang Y., Liu Y., Li Y.Z., Zhang Z. GDPF: a data resource for the distribution of prokaryotic protein families across the global biosphere. Nucleic Acids Res. 2023; 10.1093/nar/gkad869. PMC10767866 37823598

[69] Rafikova E. , Nemirovich-Danchenko N., Ogmen A., Parfenenkova A., Velikanova A., Tikhonov S., Peshkin L., Rafikov K., Spiridonova O., Belova Y. et al. Open Genes-a new comprehensive database of human genes associated with aging and longevity. Nucleic Acids Res. 2023; 10.1093/nar/gkad712. PMC10768108 37665017

[70] de Magalhães J.P. , Abidi Z., Santos D., G. A., Avelar R.A., Barardo D., Chatsirisupachai K., Clark P., De-Souza E.A., Johnson E.J. et al. Human Ageing Genomic Resources: updates on key databases in ageing research. Nucleic Acids Res. 2023; 10.1093/nar/gkad927. PMC10767973 37933854

[71] Huang K. , Liu X., Zhang Z., Wang T., Xu H., Li Q., Jia Y., Huang L., Kim P., Zhou X. AgeAnnoMO: a knowledgebase of multi-omics annotation for animal aging. Nucleic Acids Res. 2023; 10.1093/nar/gkad884. PMC10767957 37850649

[72] Li H. , Wu S., Li J., Xiong Z., Yang K., Ye W., Ren J., Wang Q., Xiong M., Zheng Z. et al. HALL: a comprehensive database for human aging and longevity studies. Nucleic Acids Res. 2023; 10.1093/nar/gkad880. PMC10767887 37870433

[73] Zhang Y. , Sun H., Zhang W., Fu T., Huang S., Mou M., Zhang J., Gao J., Ge Y., Yang Q. et al. CellSTAR: a comprehensive resource for single-cell transcriptomic annotation. Nucleic Acids Res. 2023; 10.1093/nar/gkad874. PMC10767908 37855686

[74] Xu Z. , Wang W., Yang T., Li L., Ma X., Chen J., Wang J., Huang Y., Gould J., Lu H. et al. STOmicsDB: a comprehensive database for spatial transcriptomics data sharing, analysis and visualization. Nucleic Acids Res. 2023; 10.1093/nar/gkad933. PMC10767841 37953328

[75] Wang G. , Wu S., Xiong Z., Qu H., Fang X., Bao Y. CROST: a comprehensive repository of spatial transcriptomics. Nucleic Acids Res. 2023; 10.1093/nar/gkad782. PMC10773281 37791883

[76] Harrison P.W. , Amode M.R., Austine-Orimoloye O., Azov A.G., Barba M., Barnes I., Becker A., Bennett R., Berry A., Bhai J. et al. Ensembl 2024. Nucleic Acids Res. 2023; 10.1093/nar/gkad782. PMC10767893 37953337

[77] Raney B.J. , Barber G.P., Benet-Pagès A., Casper J., Clawson H., Cline M.S., Diekhans M., Fischer C., Navarro Gonzalez J., Hickey G. et al. The UCSC Genome Browser database: 2024 update. Nucleic Acids Res. 2023; 10.1093/nar/gkad987. PMC10767968 37953330

[78] Putman T.E. , Schaper K., Matentzoglu N., Rubinetti V.P., Alquaddoomi F.S., Cox C., Caufield J.H., Elsarboukh G., Gehrke S., Hegde H. et al. The Monarch Initiative in 2024: an analytic platform integrating phenotypes, genes and diseases across species. Nucleic Acids Res. 2023; 10.1093/nar/gkad1082. PMC10767791 38000386

[79] Sondka Z. , Dhir N.B., Carvalho-Silva D., Jupe S., Madhumita K.M., Starkey M., Ward S., Wilding J., Ahmed M., Argasinska J. et al. COSMIC: a curated database of somatic variants and clinical data for cancer. Nucleic Acids Res. 2023; 10.1093/nar/gkad986. PMC10767972 38183204

[80] Liu Z. , Chen R., Yang L., Jiang J., Ma S., Chen L., He M., Mao Y., Guo C., Kong X. et al. CDS-DB, an omnibus for patient-derived gene expression signatures induced by cancer treatment. Nucleic Acids Res. 2023; 10.1093/nar/gkad888. PMC10767794 37889038

[81] Moon C.I. , Elizarraras J.M., Lei J.T., Jia B., Zhang B. ClinicalOmicsDB: exploring molecular associations of oncology drug responses in clinical trials. Nucleic Acids Res. 2023; 10.1093/nar/gkad871. PMC10767859 37811874

[82] Kumar H. , Tang L.Y., Yang C., Kim P. FusionPDB: a knowledgebase of human fusion proteins. Nucleic Acids Res. 2023; 10.1093/nar/gkad920. PMC10767906 37870473

[83] Kumar H. , Luo R., Wen J., Yang C., Zhou X., Kim P. FusionNeoAntigen: a resource of fusion gene-specific neoantigens. Nucleic Acids Res. 2023; 10.1093/nar/gkad922. PMC10767944 37870454

[84] Deng Y. , Chen P., Xiao J., Li M., Shen J., Qin S., Jia T., Li C., Chang A., Zhang W. et al. SCAR: single-cell and Spatially-resolved Cancer Resources. Nucleic Acids Res. 2023; 10.1093/nar/gkad753. PMC10767865 37739405

[85] Zhou W. , Su M., Jiang T., Yang Q., Sun Q., Xu K., Shi J., Yang C., Ding N., Li Y. et al. SORC: an integrated spatial omics resource in cancer. Nucleic Acids Res. 2023; 10.1093/nar/gkad820. PMC10768140 37811897

[86] Zhou Y. , Zhang Y., Zhao D., Yu X., Shen X., Zhou Y., Wang S., Qiu Y., Chen Y., Zhu F. TTD: therapeutic Target Database describing target druggability information. Nucleic Acids Res. 2023; 10.1093/nar/gkad751. PMC10767903 37713619

[87] Cannon M. , Stevenson J., Stahl K., Basu R., Coffman A., Kiwala S., McMichael J.F., Kuzma K., Morrissey D., Cotto K. et al. DGIdb 5.0: rebuilding the drug-gene interaction database for precision medicine and drug discovery platforms. Nucleic Acids Res. 2023; 10.1093/nar/gkad1040. PMC10767982 37953380

[88] Knox C. , Wilson M., Klinger C.M., Franklin M., Oler E., Wilson A., Pon A., Cox J. et al. DrugBank 6.0: the DrugBank Knowledgebase for 2024. Nucleic Acids Res. 2023; 10.1093/nar/gkad976. PMC10767804 37953279

[89] Zdrazil B. , Felix E., Hunter F., Manners E.J., Blackshaw J., Corbett S., de Veij M., Ioannidis H., Lopez D.M., Mosquera J.F. et al. The ChEMBL Database in 2023: a drug discovery platform spanning multiple bioactivity data types and time periods. Nucleic Acids Res. 2023; 10.1093/nar/gkad1004. PMC10767899 37933841

[90] Yin J. , Chen Z., You N., Li F., Zhang H., Xue J., Ma H., Zhao Q., Yu L., Zeng S. et al. VARIDT 3.0: the phenotypic and regulatory variability of drug transporter. Nucleic Acids Res. 2023; 10.1093/nar/gkad818. PMC10767864 37819041

[91] Zhang Y. , Liu X., Li F., Yin J., Yang H., Li X., Liu X., Chai X., Niu T., Zeng S. et al. INTEDE 2.0: the metabolic roadmap of drugs. Nucleic Acids Res. 2023); 10.1093/nar/gkad1013. PMC10767827 37930837

[92] Liu X. , Yi J., Li T., Wen J., Huang K., Liu J., Wang G., Kim P., Song Q., Zhou X. DRMref: comprehensive reference map of drug resistance mechanisms in human cancer. Nucleic Acids Res. 2023; 10.1093/nar/gkad1087. PMC10767840 37986230

[93] Schubach M. , Maass T., Nazaretyan L., Röner S., Kircher M. CADD v1.7: using protein language models, regulatory CNNs and other nucleotide-level scores to improve genome-wide variant predictions. Nucleic Acids Res. 2023; 10.1093/nar/gkad989. PMC10767851 38183205

[94] Wang Z. , Zhao G., Zhu Z., Wang Y., Xiang X., Zhang S., Luo T., Zhou Q., Qiu J., Tang B. et al. VarCards2: an integrated genetic and clinical database for ACMG-AMP variant-interpretation guidelines in the human whole genome. Nucleic Acids Res. 2023; 10.1093/nar/gkad1061. PMC10767961 37956311

[95] Cui C. , Zhong B., Fan R., Cui Q. HMDD v4.0: a database for experimentally supported human microRNA-disease associations. Nucleic Acids Res. 2023; 10.1093/nar/gkad717. PMC10767894 37650649

[96] Lin X. , Lu Y., Zhang C., Cui Q., Tang Y.D., Ji X., Cui C. LncRNADisease v3.0: an updated database of long non-coding RNA-associated diseases. Nucleic Acids Res. 2023; 10.1093/nar/gkad828. PMC10767967 37819033

[97] Sun Z.Y. , Yang C.L., Huang L.J., Mo Z.C., Zhang K.N., Fan W.H., Wang K.Y., Wu F., Wang J.G., Meng F.L. et al. circRNADisease v2.0: an updated resource for high-quality experimentally supported circRNA-disease associations. Nucleic Acids Res. 2023; 10.1093/nar/gkad949. PMC10767896 37897359

[98] Baron J.A. , Johnson C.S., Schor M.A., Olley D., Nickel L., Felix V., Munro J.B., Bello S.M., Bearer C., Lichenstein R. et al. The DO-KB Knowledgebase: a 20-year journey developing the disease open science ecosystem. Nucleic Acids Res. 2023; 10.1093/nar/gkad1051. PMC10767934 37953304

[99] Gargano M.A. , Matentzoglu N., Coleman B., Addo-Lartey E.B., Anagnostopoulos A.V., Anderton J., Avillach P., Bagley A.M., Bakštein E., Balhoff J.P. et al. The Human Phenotype Ontology in 2024: phenotypes around the world. Nucleic Acids Res. 2023; 10.1093/nar/gkad1005. PMC10767975 37953324

[100] Cao Y. , Tian D., Tang Z., Liu X., Hu W., Zhang Z., Song S. OPIA: an open archive of plant images and related phenotypic traits. Nucleic Acids Res. 2023; 10.1093/nar/gkad975. PMC10767956 37930849

[101] Dong X. , Zhao K., Wang Q., Wu X., Huang Y., Wu X., Zhang T., Dong Y., Gao Y., Chen P. et al. PlantPAD: a platform for large-scale image phenomics analysis of disease in plant science. Nucleic Acids Res. 2023; 10.1093/nar/gkad917. PMC10767946 37897364

[102] Yang Z. , Luo C., Pei X., Wang S., Huang Y., Li J., Liu B., Kong F., Yang Q.Y., Fang C. SoyMD: a platform combining multi-omics data with various tools for soybean research and breeding. Nucleic Acids Res. 2023; 10.1093/nar/gkad786. PMC10767819 37811889

[103] Kang H. , Huang T., Duan G., Meng Y., Chen X., He S., Xia Z., Zhou X., Chao J., Tang B. et al. TCOD: an integrated resource for tropical crops. Nucleic Acids Res. 2023; 10.1093/nar/gkad870. PMC10767838 37843152

[104] Chen J. , Tan C., Zhu M., Zhang C., Wang Z., Ni X., Liu Y., Wei T., Wei X., Fang X. et al. CropGS-Hub: a comprehensive database of genotype and phenotype resources for genomic prediction in major crops. Nucleic Acids Res. 2023; 10.1093/nar/gkad1062. PMC10767954 38000385

[105] Yang S. , Zong W., Shi L., Li R., Ma Z., Ma S., Si J., Wu Z., Zhai J., Ma Y. et al. PPGR: a comprehensive perennial plant genomes and regulation database. Nucleic Acids Res. 2023; 10.1093/nar/gkad963. PMC10767873 37933857

[106] Shi H. , Wu X., Zhu Y., Jiang T., Wang Z., Li X., Liu J., Zhang Y., Chen F., Gao J. et al. RefMetaPlant: a reference metabolome database for plants across five major phyla. Nucleic Acids Res. 2023; 10.1093/nar/gkad980. PMC10767953 37953341

[107] Tian Z. , Hu X., Xu Y., Liu M., Liu H., Li D., Hu L., Wei G., Chen W. PMhub 1.0: a comprehensive plant metabolome database. Nucleic Acids Res. 2023; 10.1093/nar/gkad811. PMC10767808 37819039

[108] Conroy M.J. , Andrews R.M., Andrews S., Cockayne L., Dennis E.A., Fahy E., Gaud C., Griffiths W.J., Jukes G., Kolchin M. et al. LIPID MAPS: update to databases and tools for the lipidomics community. Nucleic Acids Res. 2023; 10.1093/nar/gkad896. PMC10767878 37855672

[109] Schnider B. , M’Rad Y., El Ahmadie J., de Brevern A.G., Imberty A., Lisacek F. HumanLectome, an update of UniLectin for the annotation and prediction of human lectins. Nucleic Acids Res. 2023; 10.1093/nar/gkad905. PMC10767822 37889052

[110] Chitti S.V. , Gummadi S., Kang T., Shahi S., Marzan A.L., Nedeva C., Sanwlani R., Bramich K., Stewart S., Petrovska M. et al. Vesiclepedia 2024: an extracellular vesicles and extracellular particles repository. Nucleic Acids Res. 2023; 10.1093/nar/gkad1007. PMC10767981 37953359

